# In Situ Construction a Stable Protective Layer in Polymer Electrolyte for Ultralong Lifespan Solid‐State Lithium Metal Batteries

**DOI:** 10.1002/advs.202104277

**Published:** 2022-02-22

**Authors:** Dechao Zhang, Zhengbo Liu, Yiwen Wu, Shaomin Ji, Zhanxiang Yuan, Jun Liu, Min Zhu

**Affiliations:** ^1^ Guangdong Provincial Key Laboratory of Advanced Energy Storage Materials School of Materials Science and Engineering South China University of Technology Guangzhou 510641 China; ^2^ School of Chemical Engineering and Light Industry Guangdong University of Technology Guangzhou 510006 China

**Keywords:** dendrite‐free, interface construction, long lifespan, solid electrolyte interface, solid‐state lithium metal batteries

## Abstract

Solid‐state lithium metal batteries (SLMBs) are attracting enormous attention due to their enhanced safety and high theoretical energy density. However, the alkali lithium with high reducibility can react with the solid‐state electrolytes resulting in the inferior cycle lifespan. Herein, inspired by the idea of interface design, the 1‐butyl‐1‐methylpyrrolidinium bis(trifluoromethanesulfonyl) imide as an initiator to generate an artificial protective layer in polymer electrolyte is selected. Time‐of‐flight secondary ion mass spectrometry and X‐ray photoelectron spectroscopy reveal the stable solid electrolyte interface (SEI) is in situ formed between the electrolyte/Li interface. Scanning electron microscopy (SEM) images demonstrate that the constructed SEI can promote homogeneous Li deposition. As a result, the Li/Li symmetrical cells enable stable cycle ultralong‐term for over 4500 h. Moreover, the as‐prepared LiFePO_4_/Li SLMBs exhibit an impressive ultra‐long cycle lifespan over 1300 cycles at 1 C, as well as 1600 cycles at 0.5 C with a capacity retention ratio over 80%. This work offers an effective strategy for the construction of the stable electrolyte/Li interface, paving the way for the rapid development of long lifespan SLMBs.

## Graphical abstract

A stable solid electrolyte interface layer with multiple phases of LiF, Li_2_S*_x_*, and Li_3_N is successfully in situ formed on the electrolyte/Li surface with the ionic liquid of 1‐butyl‐1‐methylpyrrolidinium bis(trifluoromethanesulfonyl) imide as the initiator. Impressively, the LiFePO_4_/PIA‐SPE/Li solid‐state batteries exhibit admirable cyclic stabilities, and the current findings pave a new direction for fabricating long lifespan solid‐state lithium metal batteries.

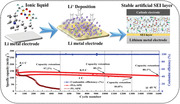

## 1 Introduction

Lightweight and rechargeable lithium (Li) metal batteries (LMBs) receive widespread attention as the candidate for high energy density energy storage systems to meet the requirements of electric vehicles and large‐scale renewable solar/wind power storage. The considerable advantages of LMBs are mainly attributed to their ultrahigh gravimetric energy density (3860 mAh g^−1^) and the lowest electrochemical potential (−3.04 V vs standard hydrogen electrode) of metallic lithium.^[^ ^1^ ^]^ Nevertheless, the direct application of the rechargeable LMBs in conventional liquid organic electrolyte configuration is inhibited, because of the serious safety issues caused by the uneven lithium deposition. In addition, the safety hazards such as the explosion and combustion of the organic liquid electrolyte caused by the short circuit of lithium dendrites, trigger the dangerous accidents and also limit the development of LMBs.^[^ ^2^ ^]^ These intractable problems are addressed through various efforts, such as Li interface modification, 3D current collector construction, and solid‐state batteries design.^[^ ^3^ ^]^ Among these strategies, replacing the conventional organic liquid electrolytes with thin Li^+^ conducting solid electrolytes in solid‐state lithium metal batteries (SLMBs) is promising to improve the safety, which is considered to be the ultimate destination of LMBs.^[^ ^4^ ^]^

Compared with liquid electrolytes, solid electrolytes (SEs) usually exhibit high mechanical strength and high thermal stability, and they can prevent the penetration of lithium dendrites and avoid explosion accidents, which gain tremendous interests for the past decades.^[^ ^5^ ^]^ Among various SEs, solid polymer electrolytes (SPEs) demonstrate a range of merits such as satisfactory plasticity and adhesion, high stability, low gravimetric density, and approachable scalability. They are considered as a kind of promising electrolyte candidate for SLMBs.^[^ ^4^, ^6^ ^]^ Nevertheless, the practical application of SPEs in SLMBs is still severely hindered by the sluggish ion transport, as well as the undesired Li/electrolyte interface reactions.^[^ ^7^ ^]^ Due to the high reducibility of lithium metal, serious irreversible reduction reactions normally occur at the Li/SPEs interface, which cause the decomposition of polymer matrix and form Li_2_O, C_2_H_4_, H_2_, etc.^[^ ^8^ ^]^ These undesirable products lead to the increase of interface impedance. Moreover, the continual corrosion reactions between the fresh Li and SPE cause a steadily increase in the interface thickness and inhomogeneous lithium surface morphology. These interface evolutions are harmful to the battery performances, leading to inferior capacity and cyclability. To address these interface issues, tremendous strategies have been proposed. Other than suppressing Li dendrite growth by exhaustedly enhancing the mechanical properties, the artificial solid electrolyte interface (SEI) layer possesses superior electronic insulation and low Li‐ion diffusion barrier, that can effectively promote the homogeneous Li deposition and protect the electrolyte/Li interface.^[^ ^9^ ^]^ Of note, in situ construction of a thin artificial protective SEI layer that is well compatible with the Li anode is particularly challenging but is an indispensable strategy to address these severe interfacial issues. To achieve this purpose, various methods have been developed. For example, by casting nanosize Cu_3_N and styrene butadiene rubber mixture on Li electrode surface, Cui et al.^[^ ^9a^ ^]^ constructed an in situ formation artificial Li_3_N SEI layer, which could suppress the formation of Li dendrites, effectively. Meanwhile, Ke et al.^[^ ^10^ ^]^ discovered that the Li‐N artificial layer could also improve the electrochemical stability of Ge‐alloy anode materials. Recently, Tao et al.^[^ ^11^ ^]^ constructed a LiF‐enriched modified interface between PEO‐based electrolyte and Li metal anode by introducing Li_2_S additive into the polymer electrolyte. The cryo‐TEM coupled with theoretical computational simulations revealed that the introduction of Li_2_S additive accelerated the formation of LiF enriched modified interface. Zheng et al.^[^ ^12^ ^]^ proposed a novel strategy to in situ form Li_2_S‐Li/electrolyte interface, and the results revealed that the Li_2_S‐modified interfacial layer could not only prevent the decomposition of PVDF matrix, but also suppress the formation of lithium dendrites. Thus, Li‐F, Li‐N, and Li‐S compounds were found as excellent interfacial components of artificial SEI, facilitating homogeneous Li deposits and protecting the SPE/Li interface in SLMBs.^[^ ^13^ ^]^ Recently, Goodenough et al. introduced Li_2_S_6_^[^ ^14^ ^]^ and Mg(ClO_4_)_2_^[^ ^15^ ^]^ into PEO‐LiTFSI based polymer electrolytes, which greatly improved the interface chemistry stability and lithium dendrite inhibition ability. Therefore, constructing a stable artificial protective layer with multiple phases between Li/SPE interface is essentially important for improving the interface stability and lifespan of LMBs.

Inspired by the aforementioned issues, herein, we first choose the ionic liquid (IL) 1‐butyl‐1‐methylpyrrolidinium bis(trifluoromethanesulfonyl)imide (PYR_14_TFSI) as a promising initiator to accelerate the generation of an artificial SEI protective layer between SPE and Li anode. The IL (PYR_14_TFSI) is selected as the initiator mainly attributed to its indispensable superiorities at least as follows: 1) the high thermal stability and low flammability can effectively avoid the risk of volatilization and leakage; 2) the high viscosity swells the coating layer to make compact contact of the SPE and Li; and 3) the addition of IL can provide additional TFSI^−^ groups, while under the participation of solvated TFSI^−^, the construction of the stable multiphase artificial SEI layer is facilitated.^[^ ^16^ ^]^ In addition, the addition of IL with lower molecular weight can be acted as a plasticizer in polymer electrolytes, which can decrease the crystallinity of polymer and speed up the polymer segmental dynamics effectively, and further improve the ionic conductivity. More addition of LiTFSI salt can also improve the TFSI^−^ group and ionic conductivity, however, the function of LiTFSI is affected by its dissociation degree in polymer electrolytes. The large amount of addition of LiTFSI salt in polymer electrolytes not only results in difficult dissolution, but also leads to the reduction of mechanical strength. Based on these considerations, the ionic liquid (PYR_14_TFSI) is selected as an ideal initiator in this work. The ester‐based biodegradable poly(*ε*‐caprolactone) and polycaprolactone diol (PCL) were selected as the solid electrolyte matrix mainly due to their processable, cost‐effective, and superior electrochemical abilities.^[^ ^7^, ^17^ ^]^ Furthermore, compared with PEO and other polymer matrices, PCL exhibits appropriate crystallinity, and possesses more C═O and C—O—C functional groups within its molecular chain, which provide more effective complex points with lithium salt and improve the Li‐ion distribution concentration along the polymer chain. In all, these advantages enhance the ionic conductivity of PCL‐based SPE. Based on these merits, PCL‐based polymer is selected as the electrolyte matrix in this work. As a consequence, by introducing the IL as the initiator, a stable artificial SEI protective layer with multiple interphases is constructed on the SPE/Li surface. The generation of the stable protective layer is strongly proved by TOF‐SIMS and ex situ X‐ray photoelectron spectroscopy (XPS) measurement techniques. Benefiting from the protection of the stable artificial protective layer, the reduction reaction of polymer electrolyte is significantly mitigated; meanwhile, the growth and propagation of lithium dendrites are suppressed effectively. As a result, ultralong‐term (more than 4500 h) stable lithium plating/stripping of the Li/Li symmetric cells is enabled, meanwhile, the cell can also cycle stably for more than 800 h at a high current density of 0.3 mA cm^−2^. The assembled Li/LiFePO_4_ SLMBs with the current IL‐modified SPEs demonstrate preferable rate performance at both 30 and 45 °C, and impressive ultralong cycle lifespan over 1300 cycles at 1 C, as well as over 1600 cycles at 0.5 C with a capacity retention ratio over 80%. As such, our work exhibits momentous implications for the design of stable SLMBs with superior cycle lifespan.

## 2 Results and Discussion

**Figure 1:**
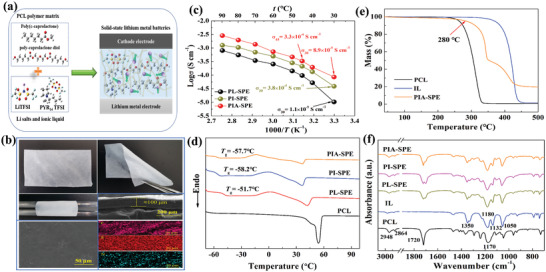
Structural and physical properties. a) Schematic diagram of the PIA‐SPE. b) Digital photographs and microstructural SEM images of PIA‐SPE membrane. c) Arrhenius plots of ion conductivity for PCL‐based electrolyte membranes with different contents. d) DSC profiles of pure PCL polymer matrix and PCL‐based electrolyte membranes. e) TGA curves of PCL polymer matrix, IL, and PIA‐SPE. f) FTIR spectra of PCL polymer matrix and PCL‐based electrolyte membranes.

In this work, the PCL‐based flexible solid electrolyte membranes were prepared by a maneuverable solution casting process, in which the electrospun nanofiber membrane (Figure S1, Supporting Information) was used as a reinforced skeleton to further enhance the mechanical strength. After the solvent evaporation, the polymer electrolyte membranes were obtained after peeling from the Teflon plate. The detailed preparation process and component optimizations are described in the Experimental Section (Supporting Information). In a typical procedure, the optimized PCL‐based SPE with the mass ratio of PCL/LiTFSI/IL/Al_2_O_3_ = 10:4:4:1 was named as PIA‐SPE. **Figure** **1**a illustrates the typical structure of IL‐modified PCL‐based polymer electrolyte (PIA‐SPE) and its lithium ions transport pathway. Figure 1b shows the morphology and microstructure of the PIA‐SPE membrane. This freestanding electrolyte film demonstrates excellent ductility and flexibility at room temperature, and it will not suffer from cracks even after several folding and coiling, indicating superior mechanical capability. As presented in scanning electron microscope (SEM) images, it could be clearly observed that the PIA‐SPE electrolyte film exhibits a flat microtopography, and the thickness of this electrolyte film is about 100 µm with a dense cross section. The cross‐sectional EDS mapping images of nitrogen (N), sulfur (S), and fluorine (F) show their uniform dispersion in the electrolyte membrane, indicating the homogeneous internal structure. By calculating the ionic conductivity in relation to temperature, the Arrhenius plots were depicted in Figure 1c. Meanwhile, the Arrhenius plots of other PCL‐based polymer electrolytes such as PL‐SPE (PCL/LiTFSI = 10:4, mass ratio) and PI‐SPE (PCL/LiTFSI/IL = 10:4:4, mass ratio) were also demonstrated in Figure 1c for comparison. The ionic conductivity of PL‐SPE and PI‐SPE electrolyte films is about 1.03 × 10^−5^ and 3.8 × 10^−5^ S cm^−1^ at ambient temperature, respectively. The improved ionic conductivity of PI‐SPE is attributed to the plasticization of the IL. After a certain amount of nano‐Al_2_O_3_ was added, the ionic conductivity of PIA‐SPE increased to 8.9 × 10^−5^ S cm^−1^, increased by eight times as compared to PL‐SPE. The improved ionic conductivity is mainly attributed to the reduced crystallinity of the PCL polymer matrix, as well as the Lewis acid interaction between Al_2_O_3_ nanoparticles and Li salts, which can promote the dissociation of salts.^[^ ^18^ ^]^ Furthermore, the ionic conductivity of PIA‐SPE increases gradually with the increase of temperature, and reaches 3.3 × 10^−4^ S cm^−1^ at 45 °C, which can meet the practical application requirements (*σ* > 10^−4^ S cm^−1^).^[^ ^19^ ^]^ In addition, the ionic conductivity of PIA‐SPE agrees well with the Vogel–Tamman–Fulcher empirical equation^[^ ^20^ ^]^ (Figure S2, Supporting Information). The determined *E*_a_ by VTF equation is 0.0465 eV. The high ionic conductivity and low activation energy may be due to the suppression of the crystallization of the PCL chains, which can reduce the polarization caused by the concentration of ions in SLMBs.^[^ ^21^ ^]^ The character of crystallinity of these PCL‐based polymer electrolytes was measured by differential scanning calorimetry (DSC). As shown in Figure 1d, the glass transition temperature (*T*_g_) of PL‐SPE is around −51.7 °C. Benefiting from the incorporation of IL and Al_2_O_3_ nanoparticles, PI‐SPE and PIA‐SPE show decreased *T_g_* of −58.2 and −57.7 °C, respectively. The decrease of the crystallinity can greatly facilitate the movement of the molecular chain segments and enhance the ionic conductivity. Furthermore, X‐ray diffraction (XRD) patterns were also measured to determine the crystallinity (Figure S3, Supporting Information). When blending with IL and nano‐Al_2_O_3_, PL‐SPE and PIA‐SPE both show decreased intensity, indicating the prevention of PCL chains from the ordered arrangement.^[^ ^22^ ^]^ Figure 1e presents the thermogravimetric analysis (TGA) results, and it is noteworthy that the IL remains stable over 300 °C before it undergoes any decomposition, which is much higher than the decomposition temperature (about 230 °C) of pure PCL polymer matrix. This excellent thermal stability indicates that a certain amount of IL addition could not damage the safety property of the SPEs. In addition, though the decomposition temperature of PIA‐SPE is slightly lower as compared with the IL, it will not decompose above about 250 °C, indicating the outstanding thermal stability of PIA‐SPE. This superior thermal stability ensures the safe use in a wide alternative temperature range. Fourier‐transform infrared (FTIR) spectroscopy was measured to verify the chemical bond transformation. Figure 1f demonstrates the FTIR spectra of the pure PCL polymer matrix and PCL‐based polymer electrolytes, in which the typical peaks located at 1720 and 1160 cm^−1^ are assigned to the C═O and C—O—C symmetrical stretching vibration of the PCL polymer matrix, respectively.^[^ ^17^, ^23^ ^]^ In the spectrum of IL, the peaks in 1350, 1180, 1132, and 1050 cm^−1^ are attributed to the S═O, CF_3_, C—SO_2_—N, and S—N—S groups of the imide anion ion TFSI^−^, respectively.^[^ ^24^ ^]^ Except for the two transforms of PIA‐SPE at 1142 and 1720 cm^−1^, indicative of the interactions by the interaction between Li^+^ and C═O, C—O—C functional groups,^[^ ^17^, ^25^ ^]^ no other obvious changes are observed, indicating the good compatibility between PCL and IL.

**Figure 2:**
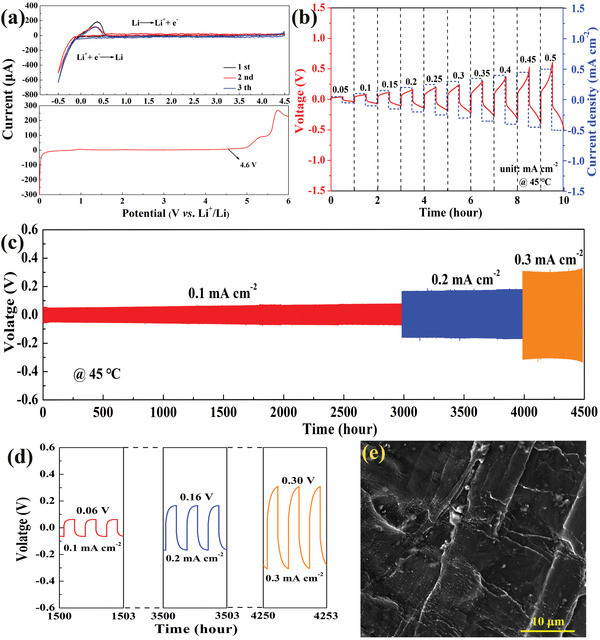
Electrochemical characterizations and interfacial stability. a) CV and LSV curves for PIA‐SPE at 45 °C. b) Galvanostatic intermittent cycling of the Li/PIA‐SPE/Li symmetric cells at step‐increased current density and 45 °C. c) Galvanostatic cycling of a Li/Li symmetric cell at 0.1, 0.2, and 0.3 mA cm^−2^ and 45 °C. d) Selected discharge/charge curves of Li/Li symmetric cell at different current densities. e) SEM images of lithium metal surface after 1000 h cycling at 0.1 mA cm^−2^.

**Figure 3:**
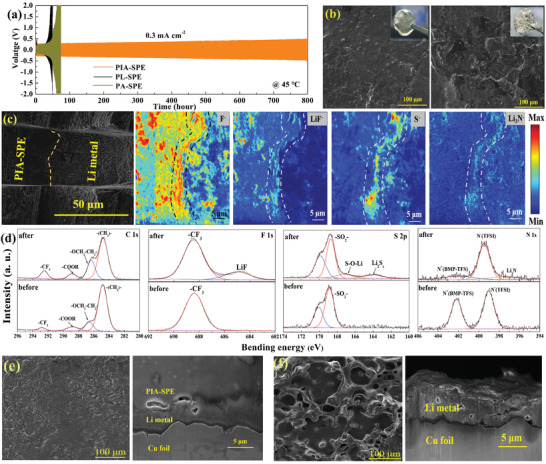
Structural evolution of the electrolyte/Li interface. a) Plating/striping performances of symmetrical Li cells with PIA‐SPE, PL‐SPE, and PA‐SPE. b) SEM images of cycled Li metal anode collected from symmetrical Li cells utilizing PIA‐SPE (left) and PL‐SPE (right). The insets are digital pictures. c) SEM image and TOF‐SIMS high lateral resolution secondary ion maps of the Li/PIA‐SPE cross section after cycling in the Li/Li cell. d) XPS spectra of C 1s, F 1s, S 2p, and N 1s of PIA‐SPE interface before and after cycling. e) Surface and cross‐section SEM images of lithium deposition morphology on Cu foil of the nonsymmetric Li/Cu cell after 1 h deposition at 0.3 mA cm^−2^ with PIA‐SPE and f) commercial liquid electrolyte (1 m LiPF_6_ in EC/DEC).

The electrochemical stable window of the PIA‐SPE membrane is determined to select proper electrode materials. Linear sweep voltammograms (LSV) and cyclic voltammetry (CV) tests in the SS/Li unsymmetrical cells are shown in **Figure** **2**a. No current peaks representing anodic reaction are observed up to 4.6 V (vs Li/Li^+^). Meanwhile, other than the lithium plating and dissolving reaction current peaks near 0 V, no other current signals relating to the oxidation are observed from the CV curves. In addition, electrochemical impedance spectra (EIS) of the Li/SS unsymmetrical cell subjected to a 4.5 V charging stage (Figure S4a, Supporting Information) and Li/Li symmetrical cell after being different aging times (Figure S4b, Supporting Information) show a slight change. All of these indicate the high stability and the possibility for matching Li metal batteries. A high lithium transference number *t* (Li^+^) can decrease the concentration polarization and promote the uniform diffusion and deposition of Li^+^, which is a crucial factor to evaluate the mobility of Li ions in SPEs.^[^ ^26^ ^]^ Figure S4c (Supporting Information) shows the polarization curve and impedance diagrams at initial and steady states. Specifically, the calculated *t* (Li^+^) of PIA‐SPE is about 0.36, which is higher than that of most polymer‐based electrolytes.^[^ ^18^ ^]^ The dynamic electrochemical stability and dendrite suppression capability between the PIA‐SPE and metallic Li anode were measured by the galvanostatic cycling test. The galvanostatic intermittent cycling of the Li/PIA‐SPE/Li symmetric cell with step‐increased current density was measured to determine the critical current density. The voltage–time plots as a function of current density from 0.05 to 0.5 mA cm^−2^ are illustrated in Figure 2b. The polarization voltage almost linearly increases as expected with increasing current density, while no short circuit occurs by the Li dendrites up to 0.5 mA cm^−2^. As shown in Figure S5 (Supporting Information), the Li/PIA‐SPE/Li symmetric cell also exhibits a stable plating/stripping cycling performance with an areal capacity of 0.1 mAh cm^−2^ at various charge/discharge current densities. The symmetric cell maintains stable cycling without an obvious increase of the polarization voltage even the current densities over 0.5 mA cm^−2^, indicting the stable interface and practicability for high power density SLMBs. Furthermore, the comparative cycle‐life tests of the Li/Li symmetric were performed by periodical plating/stripping at different current densities, demonstrated in **Figure** **3**c. The cell can perfectly work over 3000 h at 0.1 mA cm^−2^, 1000 h at 0.2 mA cm^−2^, and even can stably cycle more than 500 h at 0.3 mA cm^−2^. The typical magnified curves of the symmetric cell at different current densities are displayed in Figure 2d, in which the plating/stripping curves are stable as the current density increases from 0.1 to 0.3 mA cm^−2^ without a short circuit. In addition, the lifespan of this Li symmetric cell has exceeded the results of various SPEs reported in most of the literatures,^[^ ^27^ ^]^ indicating the effective suppression of dendrites growth by high interface compatibility with Li metal electrode. Indeed, the surface of the Li metal anode remains visibly flat (Figure 2e), as verified by SEM image of the electrodeposited Li metal even after 1000 h cycles at 0.1 mA cm^−2^. Furthermore, the lithium metal from the Li/PIA‐SPE/Li cell shows a flat surface and maintains uniform deposition layer after different plating times (Figure S6, Supporting Information). No lithium dendrite nucleation is observed, suggesting a uniform deposition of Li^+^ at the solid electrolyte/Li interface. The uniform electrodeposition of Li indicates the superior chemical and electrochemistry stabilities between Li metal and PIA‐SPE, which leads to obvious improvements in the cycle lifespan.

In an attempt to understand the influence of IL on the interface compatibility at the high current density of 0.3 mA cm^−2^, galvanostatic plating/stripping cycles were evaluated on PL and PIA‐SPE based Li symmetric cells. As demonstrated in Figure 3a, the polarization voltage of Li/PIA‐SPE/Li symmetric cell holds steady for 800 h cycles, while a significant increase appears in Li/PL‐SPE/Li and Li/PA‐SPE/Li symmetric cells after about 50 h cycles, indicating that the addition of IL exhibits an important influence on the improvement of the interface stability. Meanwhile, the morphologies of the Li metal anode are characterized and shown in Figure 3b. The surface of Li metal harvested from cells based on PIA‐SPE exhibits almost no change in color, and flat topography after 100 cycles at 0.3 mA cm^−2^. In contrast, the Li metal harvested from cells with PL‐SPE demonstrates a distinctive dimmed coloration after cycling, and obvious small moss lithium and sharp particles can be observed, which are attributed to the dead Li and Li dendrites. Such an unstable lithium deposition is significantly responsible for the inferior Li stripping/plating cycling performance. Besides, the time‐resolved EIS spectra of cycled Li/PIA‐SPE/Li symmetric cells at various time‐points (Figure S7, Supporting Information) show stable interface resistances, which is well consistent with polarization voltages during the initial 100 cycles in Figure 3a. All these results reveal that the IL exhibits a significant effect on enhancing interfacial stability. To further understand the underlying reasons, both TOF‐SIMS and ex situ XPS characterizations were used to ascertain the information about the chemical composition during the electrochemical evolution. Notably, owing to the high sensitivity to surface and elements, the TOF‐SIMS technique is used to elucidate the main chemical compositions of the Li/PIA‐SPE interface. To directly detect the distribution of the composition on the Li/PIA‐SEP solid interface, we performed a fresh and flat cross‐section interface by focused ion beam (FIB) technique on the Li/Li symmetric cell after 100 cycles (Figure 3c and Figure S8, Supporting Information). Figure 3c shows the high lateral resolution mapping of the Li/PIA‐SPE interface cross section, here, these maps provide a clear localization of F^−^, LiF^−^, S^−^, and Li_3_N^−^ at the interface. The matrix‐assisted laser desorption/ionization time of flight mass spectrometry (MALDI‐TOF‐MS) analysis was used to reveal the presence of these compounds (Figure S9, Supporting Information). These fragments sputtered off from the surface are representative chemical components of the SEI layer, indicating the addition of IL can react with the metallic lithium anode and promote the formation of an artificial SEI layer.^[^ ^13^, ^28^ ^]^ To further confirm these results, XPS depth profiling was further used to investigate the chemical environments changes at the interface. The XPS spectra of C 1s, F 1s, S 2p, and N 1s on the surface of the PIA‐SPE in the Li symmetric cell before and after cycling are given in Figure 3d. The XPS signals of the C 1s spectra are similar before and after cycling, which means that the electrochemical evolution has little effect on the chemical states of the PCL polymer matrix. However, the chemical states of F 1s, S 2p, and N 1s demonstrate obvious changes according to the XPS spectra. The binding energy located at 688.4 eV of F 1s XPS spectra of pristine PIA‐SPE is attributed to —CF_3_ of TFSI^−^.^[^ ^29^ ^]^ Notably, the new appeared peak at 684.9 eV signifies the generation of LiF.^[^ ^30^ ^]^ Specially, LiF is usually regarded as a typical component in a SEI layer to promote the ionic carrier concentration and hinder the electronic transport simultaneously, which contributes to the stable interface.^[^ ^12^, ^13^ ^]^ Except for the two S 2p double peaks presented at 168.7 and 170.1 eV, ascribed to S═O of pristine TFSI^−^, another S 2p peaks are presented at 167.3 and 163.5 eV derived from S═O after lithiation and in situ formed Li_2_S*_x_*, respectively.^[^ ^13a,b^ ^]^ As for the N 1s, the peaks around 399.1 and 402.2 eV are assigned to the N^−^ in TFSI^−^ and N^+^ in IL, respectively.^[^ ^31^ ^]^ There is an obvious change in the concentrations of the IL on the surface after cycles, while, an extra peak around 397.2 eV reveals the production of Li_3_N.^[^ ^10^, ^16^ ^]^ These assignments of the XPS spectra are summarized in Table S1 (Supporting Information). In addition, owing to the high sensitivity to the compound fragment information of TOF‐SIMS and MALDI‐TOF‐MS, the F^−^, LiF^−^, S^−^, and Li_3_N^−^ compounds can also be detected at the PL‐SPE/Li interface after cycling (Figure S10a,b, Supporting Information). However, as shown in Figure S10a (Supporting Information), the distribution of these compounds on the interface is uneven compared to that on PIA‐SPE/Li interface, which can't form a continuous stable interface protective layer. Furthermore, as shown in the XPS spectra (Figure S10c, Supporting Information) of the surface of the PL‐SPE after cycling, except that part of LiF can be detected, other compounds such as Li‐S and Li‐N compounds are not observed on the surface, indicating the inhomogeneous SEI layer between PL‐SPE and Li metal. These results strongly indicate that the modification of IL can promote the formation of the stable and homogeneous artificial SEI protective layer with passivation interphase on the Li/PIA‐SPE surface. It has been proved that the combination of organic sulfides and Li_3_N could commendably inhibit the formation of Li dendrites.^[^ ^13b^ ^]^ The effect of PIA‐SPE on lithium deposition behavior was also investigated by SEM. Figure 3e,f shows the lithium deposition morphology on Cu of the Li/Cu semisymmetric cells assumed with PIA‐SPE and commercial liquid electrolyte (1 m LiPF_6_ in EC/DEC) after a capacity of 0.3 mAh cm^−2^ Li deposition, respectively. It is clear that the morphology of Li deposition in Li/PIA‐SPE/Cu cells is uniform without lithium dendrites; meanwhile, the deposited lithium metal exhibits pyknotic cross section. On the contrary, the morphology of Li deposition on the surface of Cu foil with liquid electrolyte is random and rough along with loosened cross section. SEM images of lithium deposition morphology at the low current density of 0.1 mAh cm^−2^ demonstrate the growth pattern, as shown in Figure S11 (Supporting Information). The morphology of Li deposition presents homogeneous discoid harvested from Li/PIA‐SPE/Cu cell, but it shows an island‐like in liquid electrolyte. This phenomenon is mainly because the artificially constructed passivation layer exhibits a high exchange current density and surface energy, which increases the lithium nucleation density and promotes the uniform growth of small Li nucleus, resulting in the smooth and dense Li deposition in PIA‐SPE based cells.^[^ ^32^ ^]^ And the exchange current densities (*I*_o_) for Li deposition/stripping in various solid electrolytes calculated from Tafel plots of the Li symmetric cells are shown in Figure S12a,b (Supporting Information). The PIA‐SPE exhibits the highest *I*_o_ of 120 µA cm^−2^, which is five times larger than PL‐SPE (19 µA cm^−2^) and PA‐SPE (22 µA cm^−2^) without IL modificatory, demonstrating that the charge‐transfer kinetics is much faster in a stable SEI formed in the presence of concentrations of IL modificatory solid electrolyte.^[^ ^33^ ^]^ It is reasonable to believe that the homogeneous Li deposition and stable Li plating/stripping performance are closely related to the artificial interfacial layer, which improves the charge‐transfer kinetics, suppresses the propagation of lithium dendrites, and protects the SPEs from corrosion.

**Figure 4:**
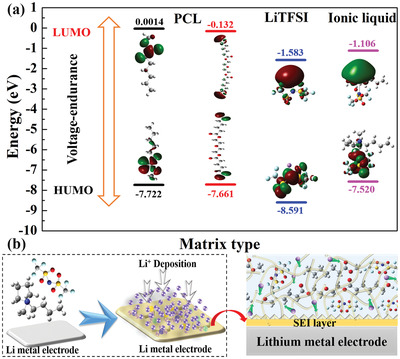
Simulation of artificial SEI protective layer formation process. a) The HOMO and LUMO levels of PCL matrix, LiTFSI, and ionic liquid. b) Schematic diagram of the formation process of the artificial SEI protective layer between PIA‐SPE and Li interface.

To verify the feasibility of the inferred chemical composition, Gauss theoretical calculation was used to confirm the experimental findings. The diagram of the orbital energy level of the main components of PIA‐SPE is shown in **Figure** **4**a. According to the calculation, the components of PCL polymer matrix exhibit a lower energy level of highest occupied molecular orbital (HOMO) (−7.72 and −7.66 eV) than commonly PEO matrix (−7.17 eV), implying the higher dielectric constant and better high‐voltage endurance of PCL matrix, which can meet the requirements for the high‐voltage cathodes.^[^ ^34^ ^]^ It is commonly known that the higher LUMO energy indicates that it is more stable and compatible with Li metal anode. The calculated LUMO of the PCL polymer matrix is much higher than that of LiTFSI and IL, indicating that LiTFSI and IL should take part in the interface reaction prior to PCL. Compared with IL, the LiTFSI delivers slightly lower LUMO energy, implying the priority of participation in interface reactions with Li metal anode. Generally, the frontier orbital energy level is determined by their own energy level of polymers matrix. But the intermolecular interactions, such as the dissociation of the lithium salt and interaction between lithium salt and polymer matrix will change the chemical environment and tune the energy‐level thermodynamically, which can enhance the activity of lithium salts participating of interfacial reaction.^[^ ^8^, ^35^ ^]^ Therefore, both the LiTFSI and IL can participate in the interfacial reaction prior to the PCL polymer matrix. It is known that the formation of SEI layer for LiTFSI‐polymer based electrolytes against lithium metal is mainly stemming from the reactivity of the TFSI^−^ anion. Thus, the addition of IL can provide more active TFSI^−^ anion, under the participation of IL and LiTFSI, a stable artificial SEI layer can be formed on the PIA‐SPE/Li surface during the cycling process. In addition, the addition of viscoelastic of IL can enhance the interface contact between lithium metal and PIA‐SPE membrane, which improves the ion transport dynamics on the interface and promotes the uniform formation of the artificial SEI layer.^[^ ^16b^ ^]^ This uniform SEI layer with multiple compounds increases the stability and promotes the homogeneous Li deposition, and this process is schematically illustrated in Figure 4b.

**Figure 5:**
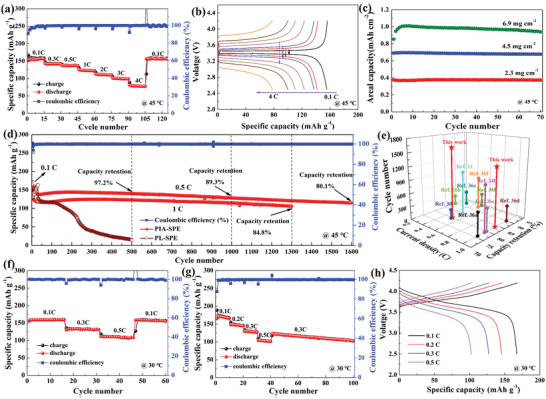
Electrochemical performances of ASLMBs. a) Rate performance obtained from the Li/PAI‐SPE/LiFePO_4_ solid‐state lithium metal batteries and b) the corresponding charge and discharge voltage profiles under different current densities at 45 °C. c) Cycle performance of Li/PAI‐SPE/LiFePO_4_ cell under high cathode areal mass loadings. d) Long‐term cycling stability at 0.5 and 1C with a temperature of 45 °C. e) The cycling performance comparison of various solid electrolytes. f) Rate performance obtained from LiFePO_4_/Li cell at 30 °C. g) Cycling and rate performances for NCM811/Li SLMB assembled with PIA‐SPE and h) corresponding charge and discharge voltage profiles under different rates at 30 °C.

With the confirmed superior stability of the PIA‐SPE against lithium metal, Li/LiFePO_4_ full cells were firstly assembled to evaluate the performance benefited from the SEI layer for the stable electrochemical performances in SLMBs. **Figure** **5**a,b displays the rate performance of Li/PIA‐SPE/LiFePO_4_ and its corresponding charge/discharge voltage profiles at various current densities (45 °C). The SLMBs deliver an excellent discharge capability of 157.2, 141.8, 137.2, and 123.5 mAh g^−1^ at 0.1, 0.3, 0.5, and 1 C (1 C = 170 mAh g^−1^), respectively. Even the current density increases to 4 C, the cell still delivers a reversible capacity of 79.8 mA h g^−1^. Moreover, when the current density switches back to 0.1 C, the specific discharge capacity recovers to 157.3 mA h g^−1^, revealing the superior rate recoverable performance. According to the galvanostatic charge/discharge voltage curves (Figure 5b), the Li/PIA‐SPE/LiFePO_4_ cell shows a typical Fe^2+^/Fe^3+^ redox couple two‐phase reaction mechanism with two flat potential plateaus at around 3.45 V.^[^ ^17b^ ^]^ Remarkably, the cell also exhibits low polarization voltages of around 8.7, 19, 28, and 43 mV at 0.1, 0.3, 0.5, and 1 C current densities, respectively. The low electrochemical polarization and stable voltage plateau at various current densities indicate the superior interface stability and integrity of the electrodes/PIA‐SPE electrolyte. Furthermore, the performance of Li/PIA‐SPE/LiFePO_4_ batteries with high cathode mass loading is also demonstrated (0.1 C current density). As displayed in Figure 5c, a high areal capacity of about 0.37, 0.68,, and 0.97 mAh cm^−2^ are obtained with the corresponding mass loading of 2.3, 4.5, and 6.9 mg cm^−2^, respectively. Meanwhile, the cycling stability and corresponding voltage profiles are shown in Figure S13a,b (Supporting Information), demonstrating the feasibility of the cell operation with practical areal capacity. To further examine the effect of this constructed SEI layer on the lifespan of SLMBs, long‐term cycling performances of the assembled Li/PIA‐SPE/LiFePO_4_ cells were investigated with 0.5 and 1 C at 45 °C. As shown in Figure 5d, after the initial few activation cycles, the SLMBs deliver a high and stable reversible capacity of about 144 mAh g^−1^ at 0.5 C. Even after 1600 cycles, a high discharge capacity over 115 mAh g^−1^ could be maintained (capacity decay of only 1.24% per 100 cycles). The charge and discharge curves only show a negligible polarization after such long‐term cycling (Figure S13c, Supporting Information). Moreover, the capacity retention ratio of the SLMBs can reach 84.9% of the highest reversible capacity under a rather high rate of 1 C, demonstrating excellent cycling stability. However, the solid state LiFePO_4_/Li cell assembled with PL‐SPE only shows a poor cycling performance, and the specific capacity decreases significantly after 100 cycles at 1 C. Notably, the Li/PIA‐SPE/LiFePO_4_ SLMBs in this work exhibit more outstanding cycling performances compared to recently reported literatures (Figure 5e and Table S2, Supporting Information).^[^ ^11^, ^26^, ^30^, ^34^, ^36^ ^]^ Furthermore, to investigate the practical application of the PIA‐SPE in ambient temperature range, Li/PIA‐SPE/LiFePO_4_ cells were also measured at 30 °C. The SLMBs could still maintain a stable capacity of 158, 133, and 110 mA h g^−1^ at 0.1, 0.3, and 0.5 C, respectively, as shown in Figure 5f and Figure S13d (Supporting Information), demonstrating the superior room temperature battery performances. In addition, these assembled SLMBs also demonstrate stable cycling performance at 0.5 C and 30 °C (Figure S13e, Supporting Information). As for a potential cathode with higher voltage and energy density, LiNi_0.8_Co_0.1_Mn_0.1_O_2_ (NCM811) was also selected as the high‐voltage cathode to verify the availability of the PIA‐SPE in the high energy‐density batteries (Figure 5g,h). Within the operating testing voltage range of 2.5–4.2 V, the solid‐state cell could deliver a high discharge capacity of about 170 mA h g^−1^ at 0.1 C and 30 °C. Even at an increased current density of 0.5 C, the battery also could output a high discharge capacity of 104 mA h g^−1^. After 100th cycles, the battery eventually displays 105 mA h g^−1^ with a capacity retention of 87.5% from the 40th cycle at 0.3 C, demonstrating the advances of the PIA‐SPE with the match of high voltage cathodes. The battery cycling performances agree very well with the observations in the Li/Li symmetric cell and the calculated prediction. The dramatically enhanced long‐term cycling stability of the SLMBs is mainly attributed to the following reasons. On the one hand, the inner better high‐voltage endurance of the PCL matrix ensured that the PIA‐SPE membrane will not occur oxidative decomposition under high voltage conditions. On the other hand, the in situ constructed stable artificial SEI layer by adding IL can facilitate the homogeneous Li deposition and protect the SPE/Li interface from corrosion significantly, which ensures the stability between lithium metal anode and PIA‐SPE membrane. In all, inner better high‐voltage endurance and well compatibility and stability between PIA‐SPE electrolyte membrane and electrodes result in the dramatically longer cycling performance of the SLMBs assembled with PIA‐SPE than the other polymer electrolytes.

**Figure 6:**
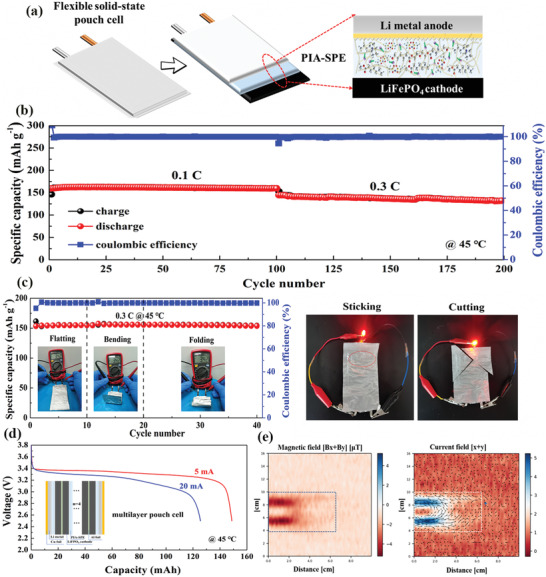
Electrochemical performance of the flexible pouch‐type ASLMBs. a) Schematic diagram of the pouch‐type solid‐state lithium metal batteries. b) Long‐term cycle stability of the pouch‐type cell at 45 °C. c) Cycling performance under different conditions of the flexible pouch‐type cell and the security test of pouch cell by lighting a LED panel under extreme circumstances. d) The tested discharge capacity profiles of the multilayer Li/LiFePO_4_ pouch cell, and the inset is the illustration of the internal structure of the pouch cell. e) The distribution of the magnetic field mapping at Bx + By and its corresponding current flow diagram of the multilayer Li/LiFePO_4_ pouch cells.

To investigate the practical application of this PIA‐SPE in flexible electronic devices, the Li/PIA‐SPE/LiFePO_4_ pouch‐type cells were also configured to demonstrate the functionality. The schematic diagram about the interior structures of the soft‐pack SLMB is presented in **Figure** **6**a. The cycling performance of this soft‐pack SLMB at 45 °C is displayed in Figure 6b, and it can be seen that the pouch‐type cell achieves a very stable reversible capacity of 160 mAh g^−1^ at 0.1 C. The corresponding voltage profiles after different cycles at 0.1 C are demonstrated in Figure S14a (Supporting Information), in which no polarization effect occurs for the soft‐pack SLMB over 100 cycles. As the current density increases to 0.3 C, the pouch‐type cell delivers a discharge capacity of 140 mAh g^−1^, with a maintained capacity of 132 mAh g^−1^ after 200 cycles. Meanwhile, this soft‐pack cell also exhibits a high specific capacity of 131 mAh g^−1^ coupling with good cycling performance at 0.5 C and 45 °C (Figure S14b, Supporting Information), indicating the good consistency of the pouch‐type and coin cell. Moreover, the evaluation of the bendability of the pouch‐type SLMBs under different conditions was conducted and shown in Figure 6c. The pouch‐type cell can cycle stably and maintain a stable output voltage under different bending conditions, indicating the promising services in flexible wearable electronics fields. Profited by the intrinsic chemical and thermal stabilities of PIA‐SPE electrolytes, reliable safety is well guaranteed even when the pouch cell is under the sticking or cut open destructive conditions in the air environment, demonstrating that the SLMBs assembled with PIA‐SPE possess superior safety in various practical conditions. Furthermore, to evaluate the practicality of the PIA‐SPE, 100 mAh‐grade Li/LiFePO_4_ pouch cell with a multilayer structure was fabricated, and the internal structure of the cell was demonstrated in Figure 6d inset. The actually measured discharge curves of the pouch cell were presented in Figure 6d. The discharge curves only show a small polarization, and a practical capacity of 148.8 and 125.5 mAh were obtained at 5 and 20 mA current density, respectively. The magnetic field mapping images were measured to determine the current distribution within the pouch cell, which was obtained from a magnetic field image measuring instrument (Figure S15, Supporting Information). Figure 6e displays the distribution of the magnetic field mapping at Bx + By and its corresponding current flow diagram. The equally distributed magnetic field indicates good current distribution within the pouch cell, and the closed‐loop of the current flow diagram indicates the current is conducted inside the pouch cell, indicating the uniform and compact internal structure, and superior stability between the PIA‐SPE and electrodes. These results demonstrate that PIA‐SPE exhibits enormous application potential in practical high energy density SLMBs.

## 3 Conclusion

In summary, based on the idea of interface design, the ionic liquid of 1‐butyl‐1‐methylpyrrolidinium bis(trifluoromethanesulfonyl) imide is used as an initiator to promote the generation of the artificial SEI protective layer between PIA‐SPE and lithium metal anode. The addition of IL provides more TFSI^−^ anion groups to participate in the construction of homogeneous and stable artificial SEI layer. A stable SEI layer with multiple phases of LiF, Li_2_S*_x_*, and Li_3_N is in situ formed on the electrolyte/Li surface, which is strongly proved by TOF‐SIMS and ex situ XPS measurements. As a result, the stable protective SEI layer ensures the Li symmetrical cell steadily plating/stripping over 4500 h. Furthermore, the SLMBs in the configuration of LiFePO_4_ and NCM811 cathodes both deliver excellent battery performances. More impressively, the LiFePO_4_/PIA‐SPE/Li solid‐state batteries exhibit admirable cyclic stabilities for over 1600 cycles and maintain a high‐capacity retention ratio over 84% at 1 C after 1300 cycles. For practical applications, the assembled LiFePO_4_/Li pouch cell demonstrates dependable safety, practical flexibility, and high discharge capacity. The present results suggest that the combination of IL with polymer electrolytes is a simple but effective strategy for the construction of the stable artificial SEI protective layer. The current work shows great possibilities for the commercial applications of SLMBs and paves a new direction for fabricating long lifespan solid‐state lithium metal batteries.

## 4 Experimental Section

The Experimental Section is available in the Supporting Information.

## Conflict of Interest

The authors declare no conflict of interest.

## Supporting information

Supporting Information

## Data Availability

The data that support the findings of this study are available from the corresponding author upon reasonable request.
