# Supplementary material for: In Situ Construction a Stable Protective Layer in Polymer Electrolyte for Ultralong Lifespan Solid‐State Lithium Metal Batteries
Source: Adv Sci (Weinh). 2022 Feb 22;9(12):2104277. doi: 10.1002/advs.202104277 (PMC9036025; doi:10.1002/advs.202104277)
Supplement: Supplementary file 1 — Supporting Information [file ADVS-9-2104277-s001.pdf]

ADVANCED SCIENCE
Open Access

Supporting Information

for *Adv. Sci.*, DOI 10.1002/advs.202104277

In Situ Construction a Stable Protective Layer in Polymer Electrolyte for Ultralong Lifespan Solid-State Lithium Metal Batteries

Dechao Zhang, Zhengbo Liu, Yiwen Wu, Shaomin Ji, Zhanxiang Yuan, Jun Liu and Min Zhu*

Supporting Information

In-situ Construction a Stable Protective Layer in Polymer Electrolyte for Ultra-long Lifespan Solid-State Lithium Metal Batteries

Dechao Zhang, Zhengbo Liu, Yiwen Wu, Shaomin Ji, Zhanxiang Yuan, Jun Liu,* and Min Zhu

D. Zhang, Z. Liu, Y. Wu, Prof. S. Ji, Z. Yuan, Prof. J. Liu, Prof. M. Zhu

Guangdong Provincial Key Laboratory of Advanced Energy Storage Materials, School of Materials Science and Engineering, South China University of Technology, Guangzhou, 510641, China

Email: msjliu@scut.edu.cn

Z. Yuan, Prof. S. Ji

School of Chemical Engineering and Light Industry, Guangdong University of Technology, Guangzhou 510006, China

Experimental section

Preparation of solid electrolyte membranes：Polycaprolactone (Mn = 68000 ~ 80000), polycaprolactone diol (average Mn ~ 2000), bistrifluoromethanesulfonimide lithium salt (LiTFSI, 99.9% purity), ionic liquid of 1-butyl-1-methylpyrrolidinium bis(trifluoromethanesulfonyl)imide, and nano Al_2O_3 (99.99%) were purchased from Aladdin. All experimental materials were stored in an Ar-filled glove box and without any further purification before use. The PIA-SPE electrolyte membranes were prepared by a universal solution-casting method. Firstly, 0.5 g polycaprolactone, 0.5 g polycaprolactone diol, 0.4 g LiTFSI, and 0.4 g IL were dissolved in 10 mL anhydrous acetonitrile, subsequently, the mixture was continuously stirred for 6 h at 60 ºC to form a slurry. Then, 0.1 g nano Al_2O_3 were added into the obtained solution and stirred for 3 h, and ultrasonic treatment for another 30 min. After that, the resulting homogeneous solution was cast onto an electrospun PAN-PVDF-HFP nanofibers membrane and heated at 60 °C for 12 h to remove the solvent. The PIA-SPE electrolyte membranes were obtained after peeling off from the polytetrafluoroethylene mold. As for comparison, the PL-SPE (with 0.5 g polycaprolactone, 0.5 g polycaprolactone diol, and 0.4 g LiTFSI), PA-SPE (with 0.5 g polycaprolactone, 0.5 g polycaprolactone diol, 0.4 g LiTFSI and 0.1 g nano Al_2O_3), PI-SPE (with 0.5 g polycaprolactone, 0.5 g polycaprolactone diol, 0.4 g LiTFSI and IL) were also prepared according to the same method. All these processes were carried out in an argon-filled glove box. The electrospun PAN-PVDF-HFP (PAN: PVDF-HFP = 1: 1 mass ratio) nanofiber membrane was prepared by the

electrospinning technique.

Materials characterizations: The crystal structure characterization of the samples were identified *via* X-ray diffraction (XRD) with PANalytical Empyrean XRD with Cu–Kα radiation. X-ray photoelectron spectroscopy (XPS) spectra were performed on a Thermo K-Alpha XPS spectrometer equipped with a monochromatic Al-Kα X-ray source. Scanning electron micrographs (SEM) and energy dispersive spectral (EDS) mapping images were obtained with a TESCAN GAIA3 field-emission scanning electron microscope equipped with energy dispersive spectrometry. The existence of atoms and molecular fragments was detected by time-of-flight secondary ion mass spectrometry (TOF-SIMS) (ION-TOF TOF-SIMS Ⅳ) measurements with a pulsed 30 KeV gallium (Ga) liquid metal ion beam source and negative mode. Meanwhile, the atoms and molecular fragments with negative charge can be captured by the probe, and the matrix-assisted laser desorption/ionization time of flight mass spectrometry (MALDI-TOF-MS) of different atoms and molecular fragments was obtained according to the mass spectral analyses. The thermal stability was tested by differential scanning calorimetry (DSC) (TA-Q20 differential scanning calorimeter) and thermogravimetric analysis (TGA) (Q50 thermogravimetric analyzer) with a heating scan rate of 5 °C min^{-1} in Ar atmosphere. The chemical bonds transformations were recorded by Fourier transform infrared spectroscopy (FT-IR) (FT-IR Nicolet IS50 instrument). The orbital energy levels of the main components of PIA-SPE were calculated according to the principle of the Gauss theorem.

Electrochemical characterizations: The electrochemical performances of polymer

electrolytes were measured by a Gamry Interface 1000 electrochemical workstation. The temperature dependence ionic conductivity from 30 to 90 °C was recorded by the electrochemical impedance spectroscopy (EIS) in the SS/SS symmetrical cell with the frequency range of 0.1 Hz to 1 MHz and AC amplitude of 10 mV. The activation energy E_a for the Li^+ conduction in PIA-SPE is calculated according to the Vogel−Tamman−Fulcher (VTF) equation as follow:

$$\sigma = AT^{-1/2} \exp(-E_a/k_b\,(T-T_0)) \quad (1)$$

Where A is the pre-exponential factor, k_b presents the Boltzmann constant, T_0 is a parameter correlated to the glass transition temperature (T_g), σ is the ionic conductivity. The electrochemical stability of the electrolyte was conducted by cyclic voltammetry (CV) and linear sweep voltammetry (LSV) measurements of the SS/Li unsymmetrical cell with the scan rate of 1 mV s^{-1} at 45 °C, where stainless steel (SS) was used as the working electrode and Li metal as the reference electrode. The interface stability against Li metallic electrodes of the electrolyte membrane was measured by the lithium periodic stripping/plating tests of the Li/Li symmetric cells 45 °C. Direct-current (DC) polarization combined with AC impedance in a Li/Li symmetric cell was applied to examine the lithium ions transference number t (Li^+), which can be estimated according to the following Equation:

$$t\,(Li^+) = I_s(\Delta V - I_0R_0)/I_0(\Delta V - I_sR_s) \quad (2)$$

where ΔV is the applied DC potential (10 mV), I represents the direct current, R is the charge-transfer resistance of the passivation layers. The subscripts of "*0*" and "*S*" represent the initial and steady state, respectively. The exchange current density (I_0)

was calculated using a linear fit of Tafel plots (from 150 mV to 100 mV), and the Tafel plots were in turn obtained from the linear sweep voltammetry for Li symmetric cells employed a fixed sweep rate of 0.1 mV s^{-1} with the voltage range from -300 mV to 300 mV.

SLMBs full-battery assembly and electrochemical measurements: The mixture of $LiFePO_4$ (supplied by Dynanonic Ltd.) or $LiNi_{0.8}Mn_{0.1}Co_{0.1}O_2$ cathode materials, PVDF binder, and conductive carbon black (8:1:1 in weight ratio) were stirred continuously in N-Methyl pyrrolidone (NMP) solvent to form a homogeneous slurry, then the slurry was cast onto the aluminum foil and dried in vacuum at 80 °C for 12 h. The mass loading of active materials was about 1.8-2.5 mg cm^{-2}. The $LiFePO_4$ cathode with high mass loading was prepared by the same method. In order to infiltrate the surface and enhance interfacial ion transport, 10 µL polymer electrolyte mixture solution was cast onto the cathode and dried at 60 °C before battery assembly. CR 2032 coin-type cells were assembled in an Ar-filled glovebox with Li metal foils as the anode. The galvanostatic charge/discharge tests were recorded on a LAND CT2001A testing system. The magnetic field distribution mapping images were measured by a B-LAB magnetic field image measuring instrument (DENKweit Corporation, Germany).

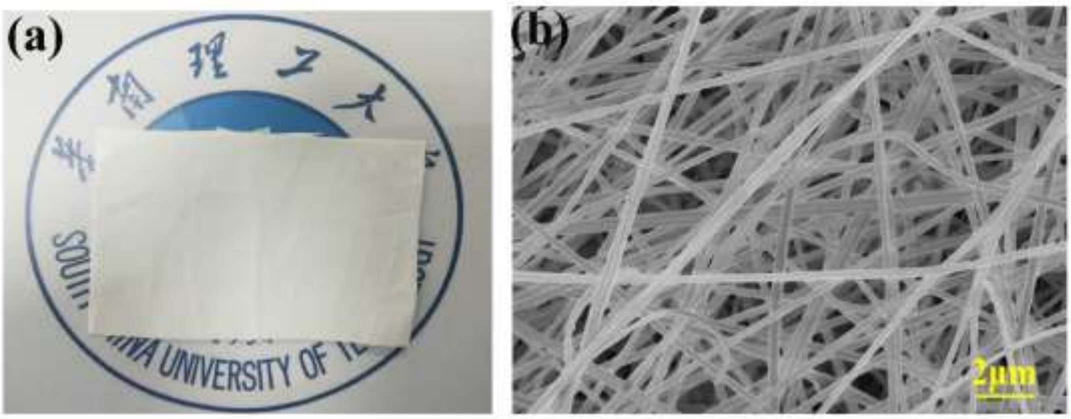

Figure S1. Optical photograph and SEM image of the electrospun nanofiber membrane.

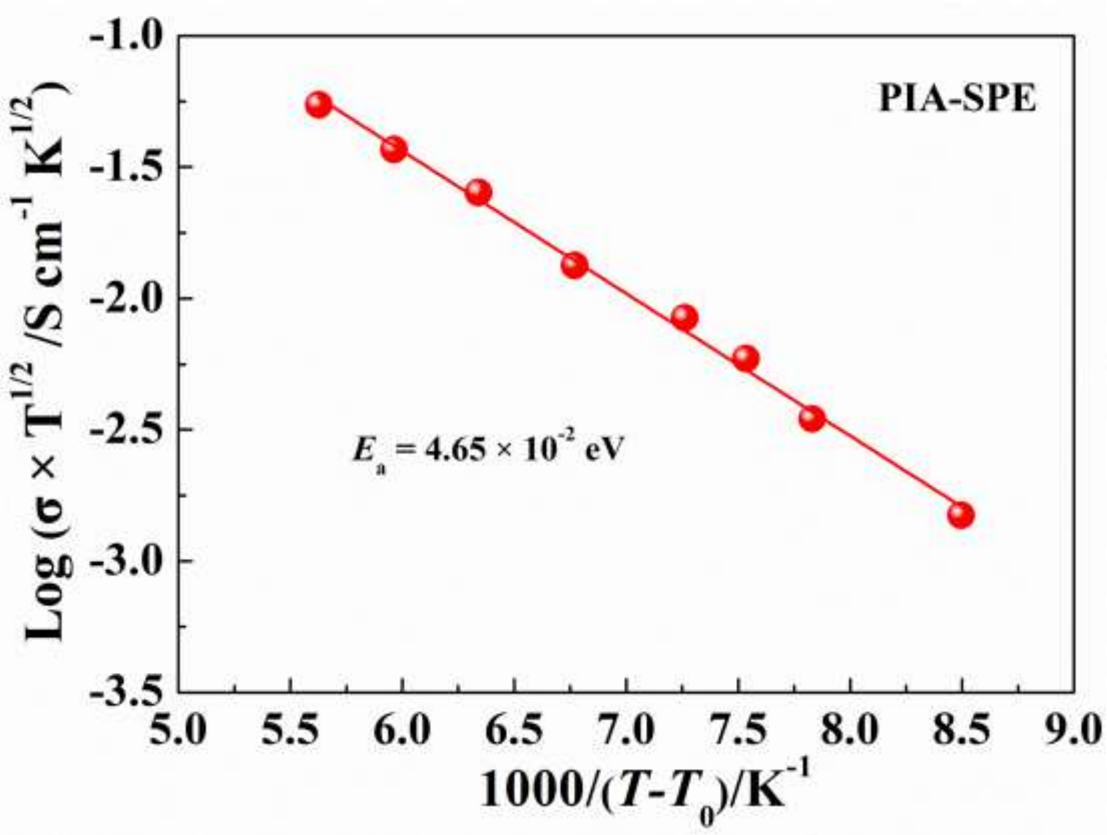

Figure S2. The VTF fitting of ionic conductivity data with different temperatures for PIA-SPE.

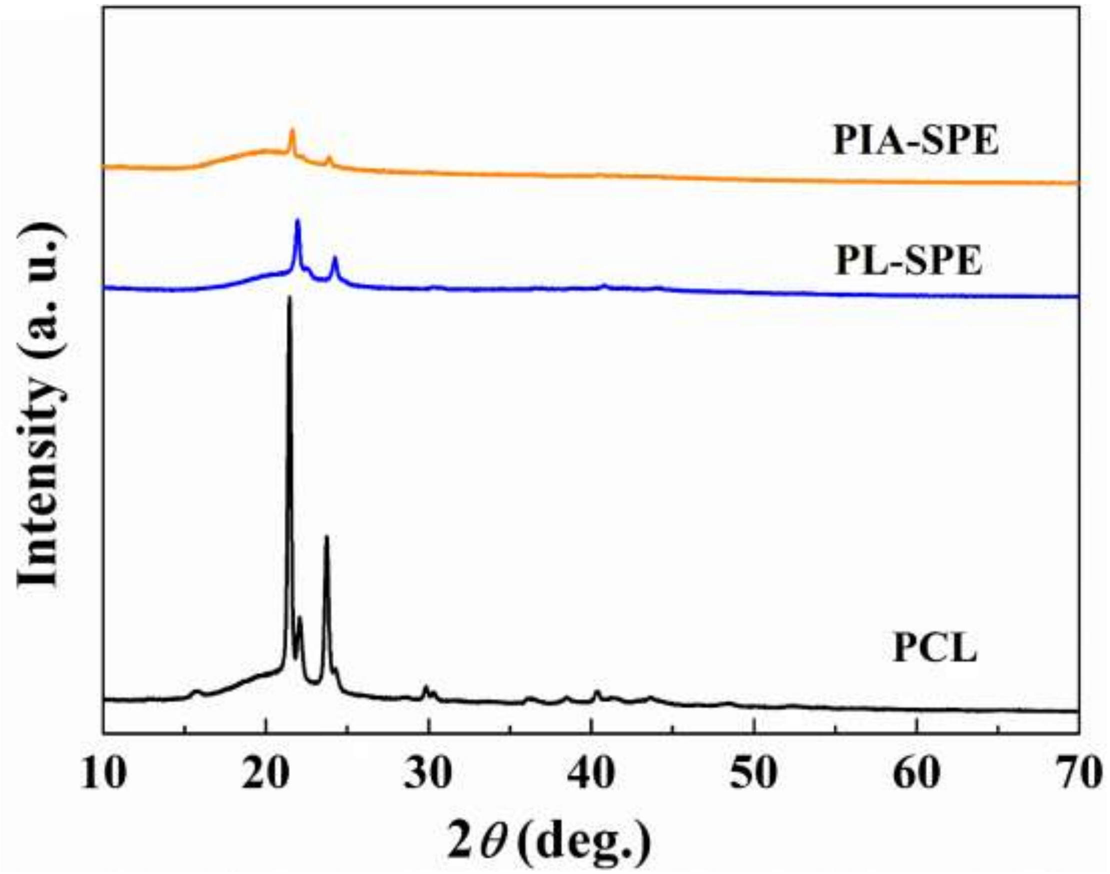

Figure S3. XRD patterns of PCL and PCL-based polymer electrolytes (PL-SPE and PIA-SPE).

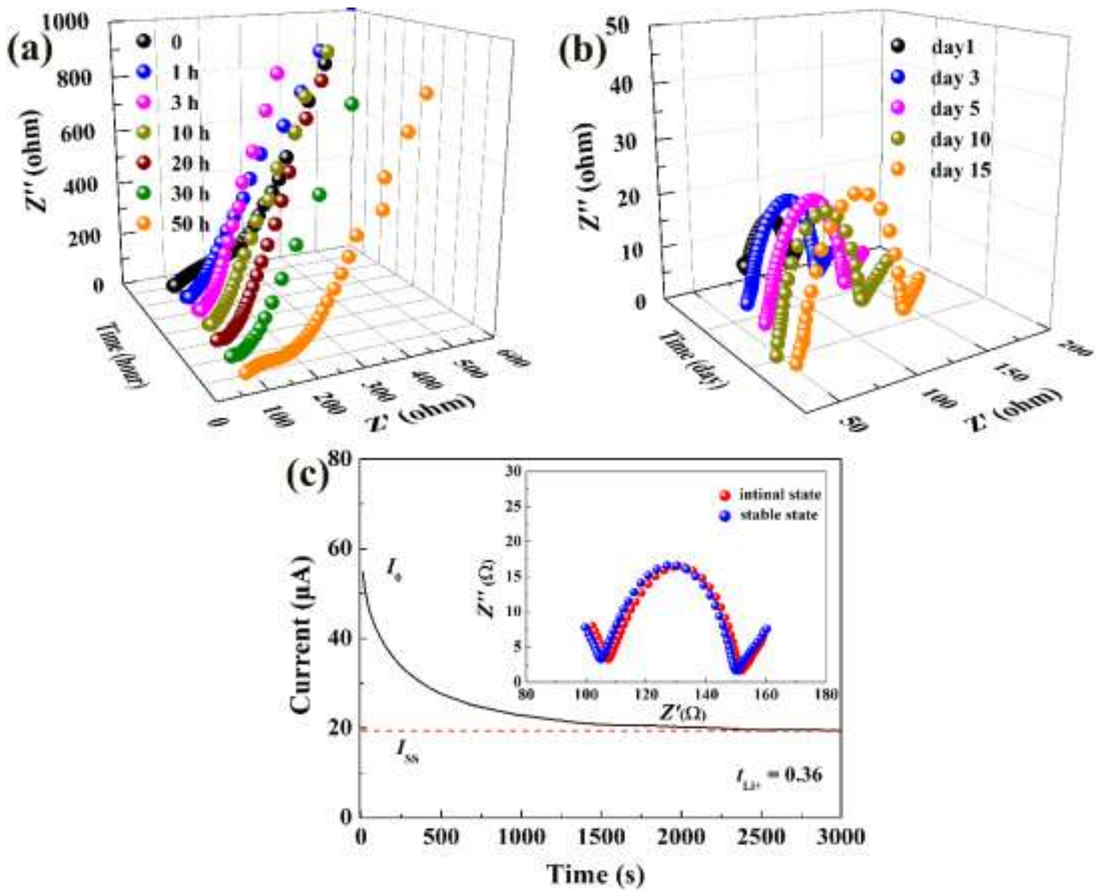

Figure S4. (a) Impedance spectra of SS/PIA-SPE/Li unsymmetrical cell at bias voltage 4.5 V. (b) Nyquist plots of the Li/PIA-SPE/Li symmetric cell at different storage times. (c) Polarization curve and initial and steady state impedance spectra for PIA-SPE electrolyte membrane at 45 ºC.

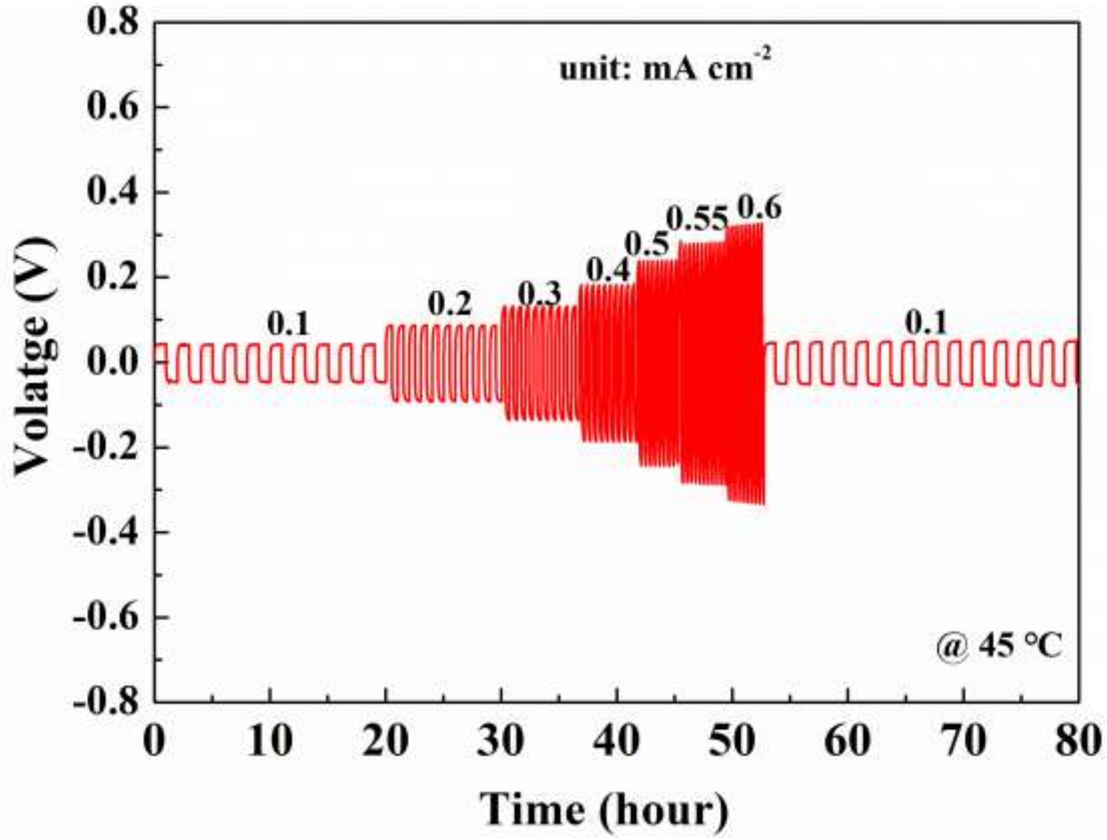

Figure S5. The galvanostatic cycling profile of the Li/PIA-SPE/Li cell at various current densities with an areal capacity of 0.1 mAh cm^{-2} and measured at 45 °C.

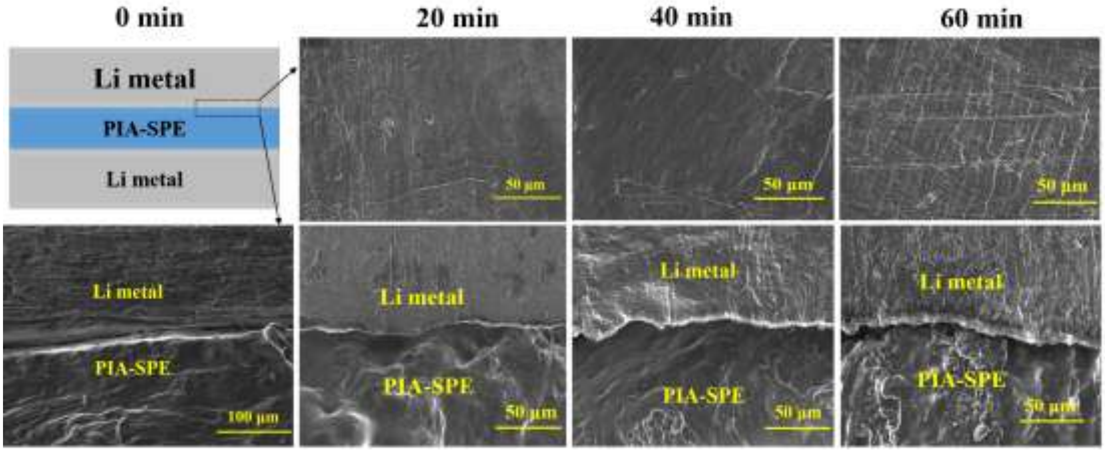

Figure S6. Surface and cross-sectional SEM images of Li/PIA-SPE/Li cell after different Li plating times.

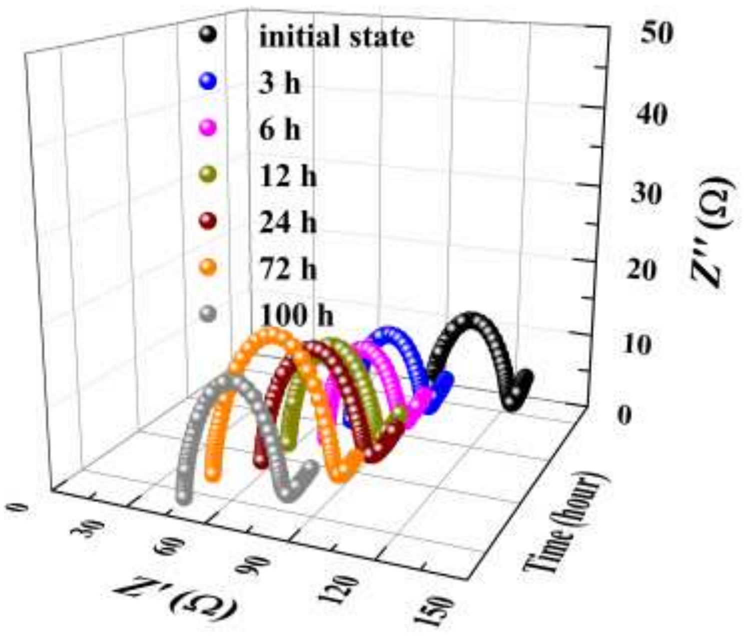

Figure S7. Time-dependent EIS spectra of symmetrical Li/PIA-SPE/Li cell at the current density of 0.3 mA cm^{-2} and 45 ºC.

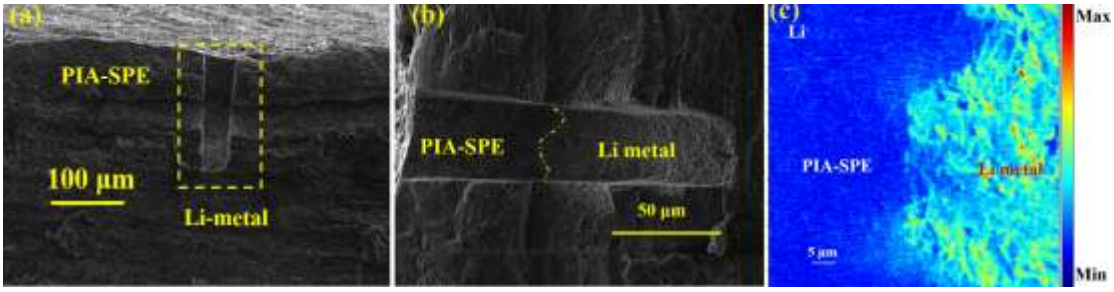

Figure S8. (a,b) SEM images of the Li/PIA-SPE cross-section after cycling in the Li/Li. (c) TOF−SIMS high lateral resolution secondary ion maps of Li^- on the cross-section.

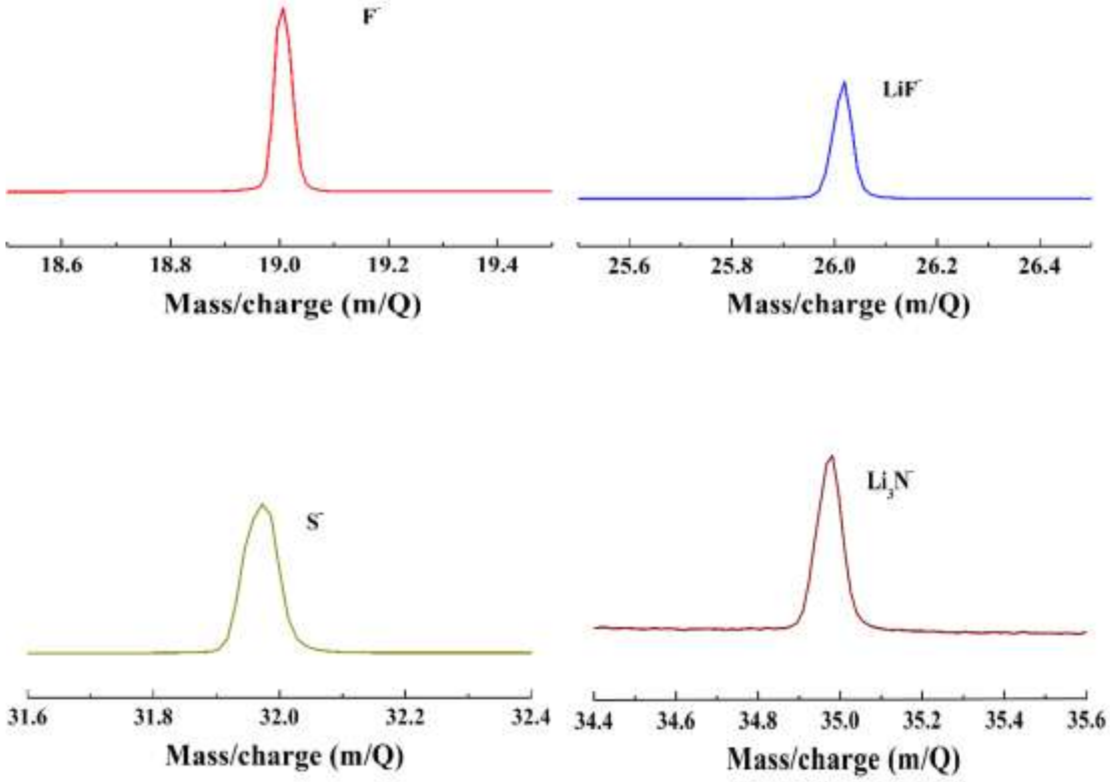

Figure S9. MALDI-TOF-MS analysis of the products of F^-, LiF^-, S^- and Li_3N^-.

Table S1. The assignments of XPS spectra of PIA-SPE after Li/Li cycling.

XPS signals	Binding energy (eV)	Description of bands
C 1s	284.8	-CH_2-
	286.6	-OCH_2-CH_2-
	284.4	-COOR-
	292.8	-CF_3 of $TFSI^-$ anionic group
F 1s	688.4	-CF_3 of $TFSI^-$ anionic group
	684.9	the generation of LiF
S 2p	168.7	S=O of pristine $TFSI^-$
	170.1	
	167.3	S-O-Li derives from S=O after lithiation
	163.5	in situ formed Li_2S_x
N 1s	391.1	N^- in $TFSI^-$ anionic group
	402.2	N^+ in IL
	397.2	the production of Li_3N

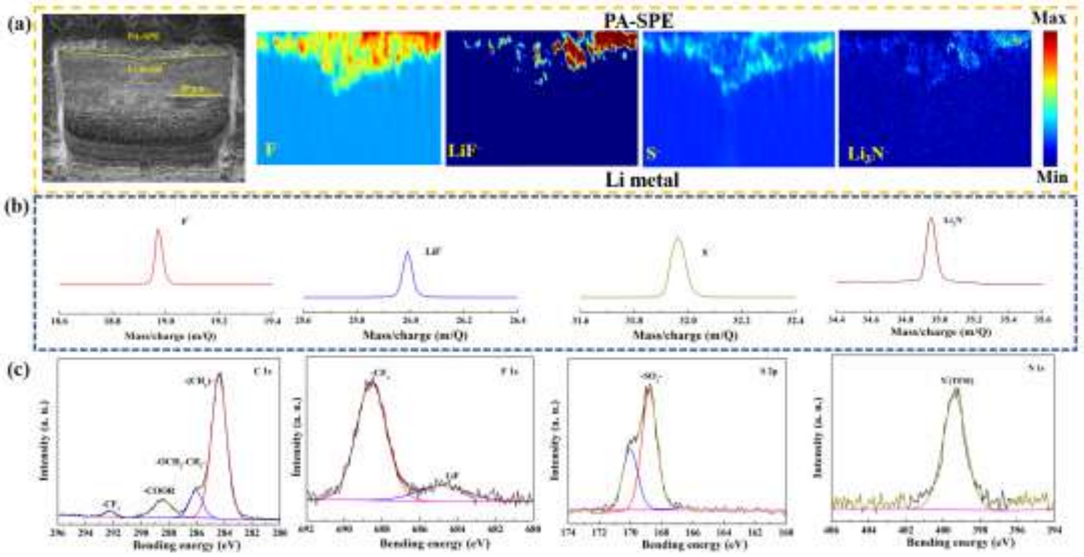

Figure S10. (a) SEM image and TOF−SIMS high lateral resolution secondary ion maps of the Li/PA-SPE cross-section after cycling in the Li/Li cell. (b) MALDI-TOF-MS analysis of the products of F^-, LiF^-, S^- and Li_3N^-. (c) XPS spectra of C 1s, F 1s, S 2p, and N 1s of PA-SPE interface after cycling.

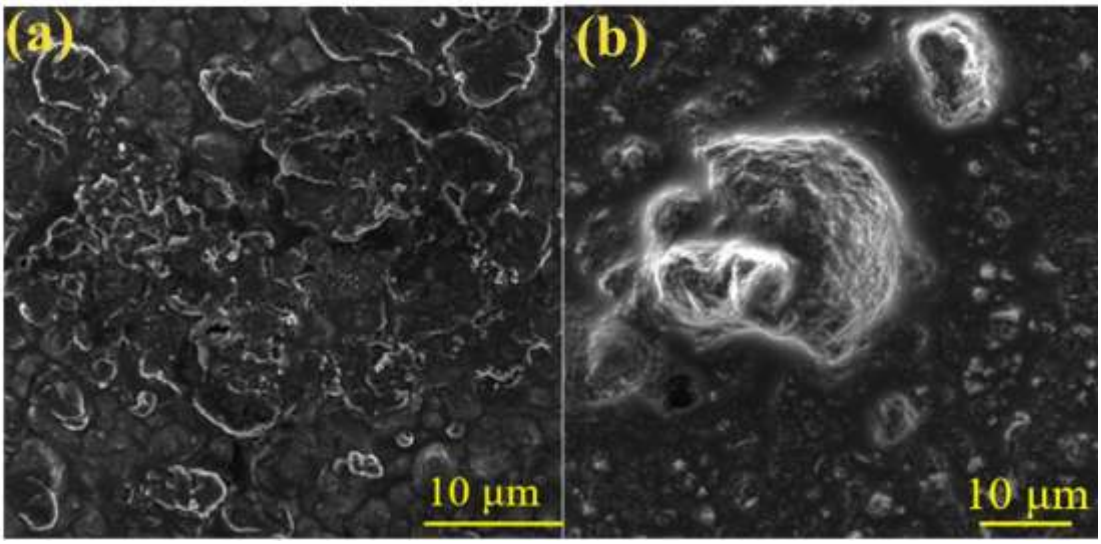

Figure S11. SEM images of lithium deposition morphology on Cu foil of the unsymmetric Li/Cu cell at 0.1 mAh cm^{-2} with PIA-SPE (a) and the commercial liquid electrolyte of 1M $LiPF_6$ in EC/DEC (b).

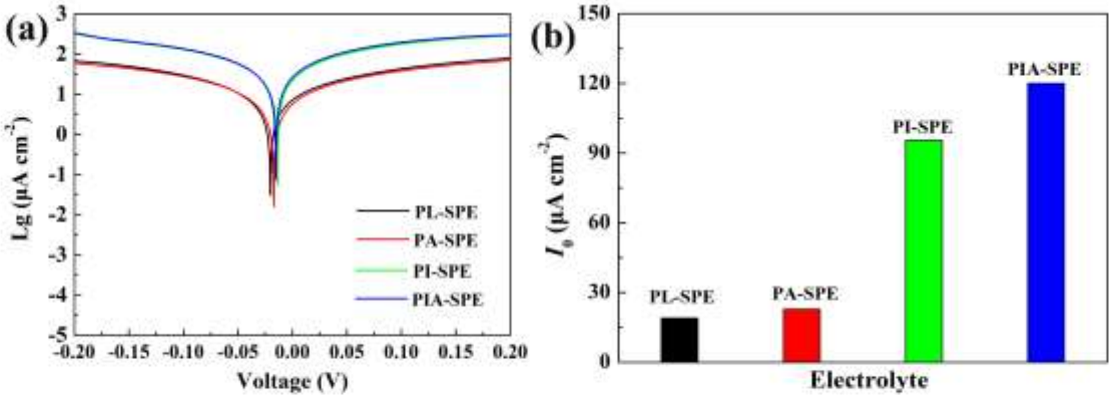

Figure S12. (a) Tafel plots obtained from cyclic voltammetry measurements. (b) Comparison of exchange current densities (I_0).

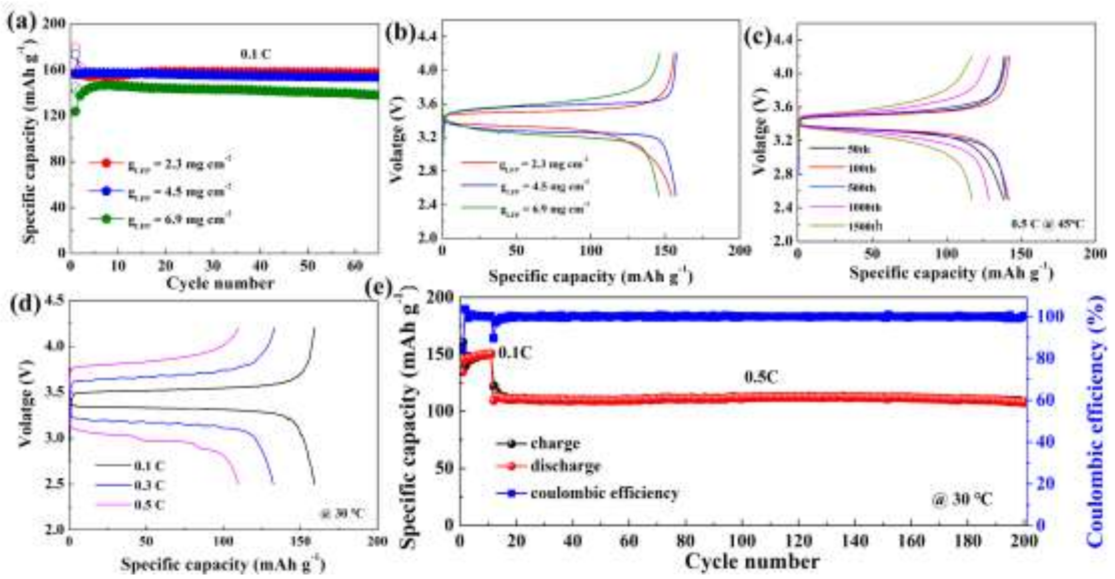

Figure S13. (a) Cycling stabilities of $LiFePO_4$/PIA-SPE/Li cell with different cathode areal mass loadings at 0.1 C and 45 ºC. (b) The corresponding charge and discharge voltage profiles. c Charge and discharge curves of different cycles obtained from $LiFePO_4$/PIA-SPE/Li cell at 0.5 C and 45 ºC. (d) Charge and discharge curves under different rates at 30 ºC. (e) Cycling stability obtained from $LiFePO_4$/PIA-SPE/Li cell at 0.5 C and 30 ºC.

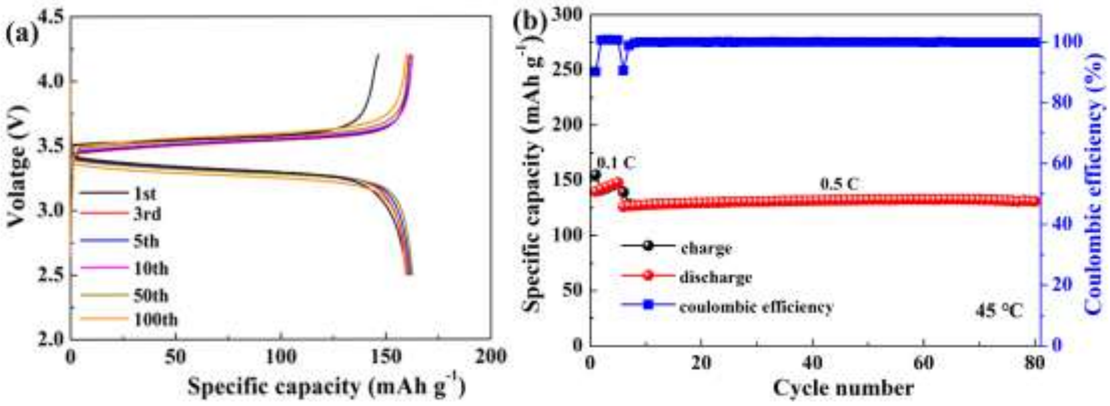

Figure S14. (a) The charge and discharge voltage profiles of $LiFePO_4$/PIA-SPE/Li soft-pouch cell after different cycles. (b) The cycling performance of $LiFePO_4$/PIA-SPE/Li soft-pouch cell at 0.5 C and 45 ºC.

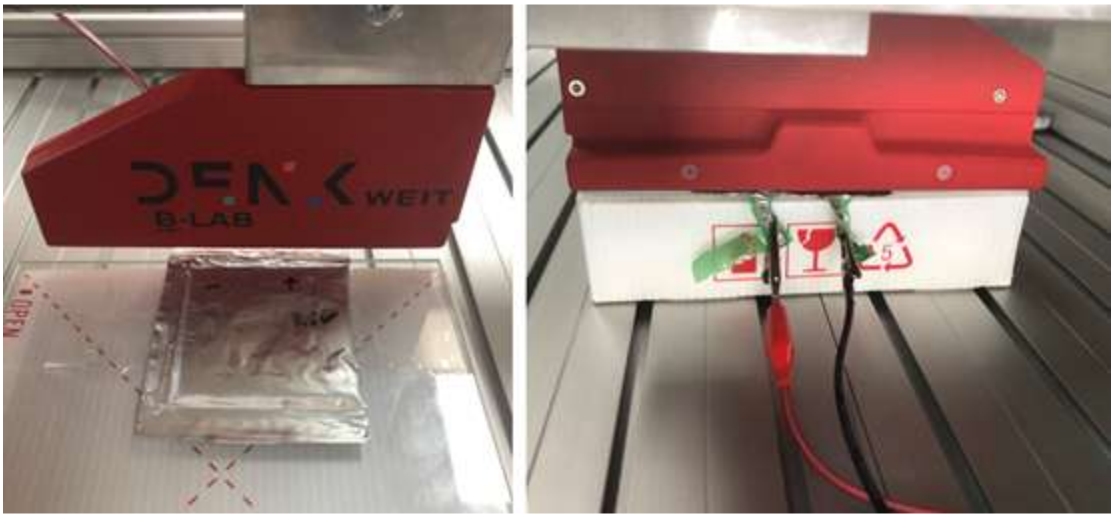

Figure S15. The optical images of the magnetic field distribution mapping image tests measured by a B-LAB magnetic field image measuring instrument.

Table S2. Comparison of the electrochemical performances between $LiFePO_4$/PIA-SPE/Li SLMB and other recent works.

Solid-state electrolytes	Electrodes	Working temperature (ºC)	Current density (C)	Specific capacity (mA h g^{-1})	Cycle number	Capacity retention rate	References
PEO-$LiClO_4$-LATP	$LiFePO_4$	80	1	109.3	500	76.3	*J. Phys. Chem. C*, **2018**, 122, 9852-9858
PTFE-LLZTO-SN	$LiFePO_4$	25	0.2	152.7	200	90.3	*Adv. Energy Mater.*, **2020**, 1903376
PEOm-$Li_{21}Si_5$	$LiFePO_4$	45	0.5	111.3	200	79.5	*Adv. Mater.*, **2021**, e2004711
PEO-LiTFSI-Li_2S	$LiFePO_4$	50	0.5	140	1000	85	*Adv. Mater.*, **2020**, 2000223
LLZTO-HCSE	$LiCO_2$	25	1	96.7	1000	79	*Adv. Funct. Mater.*, **2020**, 2006381
LAGP-PEO	$LiFePO_4$	60	0.3	138.8	300	93.3	*Nano Energy*, **2019**, 60, 205-212
PI-PEO-LiTFSI	$LiFePO_4$	60	0.5	138	300	95	*Nat. Nanotechnol.*, **2019**, 14, 705-711

PEO-LSTZ	$LiFePO_4$	45	-	123	350	90	*Proc. Nat. Acad. Sci. USA*, **2019**, 116, 18815-18821
PEO-SN-LiTFSI	$LiFePO_4$	25	0.5	135.7	750	93	*Adv. Funct. Mater.*, **2020**, 30, 2007172
PEO_{18}-LiTFSI-LLZO-SN	$LiFePO_4$	60	1	108.8	500	80	*J. Electrochem. Soc.*, **2018**, 165, A3558-A3565
**This work**	**$LiFePO_4$**	**45**	**0.5**	**115.3**	**1600**	**80.1**	
			**1**	**106**	**1300**	**84.8**	
